# Clinical Outcomes of Intraoperative Radiation Therapy for Extremity Sarcomas

**DOI:** 10.1155/SRCM/2006/91671

**Published:** 2006

**Authors:** Quy N. H. Tran, Anne C. Kim, Alexander R. Gottschalk, William M. Wara, Theodore L. Phillips, Richard J. O'Donnell, Vivian Weinberg, Daphne A. Haas-Kogan

**Affiliations:** ^1^Department of Radiation Oncology, Comprehensive Cancer Center, 1600 Divisadero Street, Suite H1031, San Francisco, CA 94143-1708, USA; ^2^Department of Orthopaedic Surgery, Comprehensive Cancer Center, University of California at San Francisco, 1600 Divisadero Street, 4th Floor, San Francisco, CA 94115-1939, USA; ^3^Biostatistics Core, Comprehensive Cancer Center, University of California at San Francisco, 1600 Divisadero Street, Suite H1031, San Francisco, CA 94143-1708, USA

## Abstract

*Purpose*. Radiation of extremity lesions, a key component of limb-sparing therapy, presents particular challenges, with significant risks of toxicities. We sought to explore the efficacy of intraoperative radiation therapy (IORT) in the treatment of soft tissue sarcomas of the extremities. *Patients*. Between 1995 and 2001, 17 patients received IORT for soft tissue sarcomas of the extremities. Indications for IORT included recurrent tumors in a previously radiated field or tumors adjacent to critical structures. *Results*. Gross total resections were achieved in all 17 patients. Two patients experienced locoregional relapses, six patients recurred at metastatic sites, and one patient died without recurrence. Thirty-six month estimates for locoregional control, disease free survival, and overall survival were 86%, 50%, and 78%, respectively. IORT was extremely well tolerated, with no toxicities referable to IORT. *Conclusions*. For patients with soft tissue sarcomas of the extremities, IORT used as a boost to EBRT provides excellent local control, with limited acute toxicities.

## INTRODUCTION

Soft-tissue sarcomas (STSs) are relatively uncommon tumors, representing 1% of adult and 7%–15% of pediatric malignancies. They are a relatively heterogeneous group of cancers that occur anywhere in the body, with 60% arising in the extremities. There are at least 30 distinct histologic subtypes of sarcomas that are further defined by grade [1]. Such diversity may present a clinical challenge, but for treatment purposes, most soft-tissue sarcomas are grouped together. Important prognostic factors, reflected in the newly revised American Joint Committee on Cancer (AJCC) staging system, include histologic grade, relationship to fascial planes, and size of the primary tumor [2].

In the past, sarcomas arising in the extremities were frequently treated with amputation, as limited resections resulted in poor local control rates. In the past two decades, limb-salvage approaches have drawn interest and attention, particularly with the recent advent of multimodality therapy. Treatment regimens that combine surgery, chemotherapy, and radiation have allowed treating physicians to maintain function without compromising disease control [3–10]. Limb-sparing therapy is therefore becoming the standard of care [11, 12], with less than 10% of patients currently undergoing amputation as primary therapy. With modern approaches, local control rates using limb-sparing techniques exceed 75% for primary extremity lesions [11, 13].

Recently, intraoperative radiation therapy (IORT) has been gaining favor. At our institution, IORT consists of a single fraction of radiation, using electrons, administered at the time of resection. Critical structures can be visualized and manipulated to avoid dose-limiting toxicities to normal structures, while a higher dose can be applied to residual tumor volume and sites at high risk for microscopic disease. Retrospective studies of various malignancies have suggested that IORT may improve local control compared to standard radiotherapy [14–18].

In conjunction with external beam radiation therapy (EBRT), IORT contributes to higher total doses of radiation to the tumor bed, while limiting long-term side effects. Thus, IORT allows for potentially higher local control rates while reducing radiation toxicities [14–20]. Early clinical trials utilizing IORT have reported favorable local control rates with few patients experiencing IORT-related side effects [21, 22].

Radiation has a well-established role in the treatment of sarcomas, and we sought to enhance local control without increasing radiation-induced toxicities by utilizing IORT as a boost. Specifically, in this study, we sought to determine the rates of local control, disease-free survival (DFS), overall survival (OS), and acute toxicities in patients who had received limb-sparing therapy with IORT for sarcomas of the extremities at the University of California, San Francisco (UCSF).

## PATIENTS

In this retrospective study, we report on 17 patients with soft-tissue extremity sarcomas who were treated with limb-sparing therapy, including IORT, between 1995 and 2001. Details of surgery, radiation, chemotherapy, as well as imaging studies and pathological diagnoses were acquired. Follow-up duration was calculated from the date of IORT until the last known or documented visit or the date of death. Patients were staged according to the AJCC staging system for sarcomas that incorporates histologic grade, tumor size, lymph node status, and metastases [2]. All patients in this study had gross total resections (GTRs) with IORT at the time of surgery.

Intraoperative radiation therapy boosts for extremity sarcomas have been used at UCSF since 1995, but prior to 1997, IORT was delivered in a suite in the Radiation Oncology Department; patients were brought to the room at the time of or within 2 days of surgery. Since 1997, patients have received IORT at the time of primary resection within the operating room, by means of a dedicated mobile linear accelerator [23]. Electron beams were delivered through lucite or aluminum cones 3–10 cm in interior diameter. Beam energies ranging from 4 MeV to 12 MeV were used to limit the depth of the absorbed dose to the areas at risk, while encompassing the target volumes within the 90% isodose line. The median dose was 12.5 Gy (range 12–15 Gy) delivered in a single fraction. One to two separate IORT fields were used to cover the entire target volume. The surgeon and radiation oncologist made final decisions at the time of surgery regarding the areas at risk for microscopic residual disease. The target volume was the tumor bed as determined by preoperative imaging, operative findings, and, when necessary, intraoperative frozen sections. Shielding of normal tissues not at risk for disease was accomplished by physical manipulation to exclude them from the radiation field, or by lead sheets.

The indications for IORT included recurrent tumors within a previously radiated field, or tumors where margin status was in question, due to close proximity of critical structures, such as nerves or vessels. Patients with tumors close to critical structures were treated with IORT as a boost, rather than preoperative radiation, because of the increased wound complications seen with preoperative radiation therapy [24]. This study was approved by the Committee on Human Research, University of California, San Francisco, Institutional Review Board.

## METHODS

Descriptive statistics were used to characterize these patients with extremity sarcomas. A single group of 17 patients was analyzed, and therefore no statistical comparisons of subsets were performed. Between 1995 and 2001, 61 patients total were treated with radiation for soft-tissue sarcomas of the extremities at our institution. The Kaplan-Meier product limit method was used to estimate the probabilities of local control, DFS, and OS. Survival was measured from the date of IORT treatment until the date of death or date of last contact, if the patient was still alive. DFS was measured from the date of IORT treatment until the date of failure or death or date of last contact if the patient was last known to be disease free. Local failure was defined as documented disease within the radiation field or in the primary site, and regional failure was defined as disease recurrence directly adjacent to the original site of disease or in adjacent lymph node groups.

## RESULTS

Patient characteristics are summarized in Table 1. High-grade lesions were noted in all stage II patients, while the single stage I patient had a low-grade lesion. Six stage III patients and one stage IV patient had high-grade lesions. No grade was noted on pathology reports of one stage III patient and two stage IV patients. All patients had GTRs, but 8 patients had previous attempts at resections. Following GTRs, 6 patients had positive surgical margins. Ten of 17 patients received chemotherapy as part of their initial treatment. Although it is our institutional policy to administer neoadjuvant chemotherapy for most high-grade sarcomas in adults (usually a doxorubicin- or ifosfamide-based regimen), the remaining patients did not receive chemotherapy due to either low-grade histology, advanced age, or poor Karnofsky performance status.

IORT was administered at the time of original presentation in 16 patients and at the time of tumor recurrence in 1 patient. Thirteen of 17 patients received postoperative external beam radiation therapy, with a median dose of 50.3 Gy (range 43.2–61.2 Gy) and a median number of 26 fractions (range 20–33 fractions). Treatment was completed within a median time of 76 days (range 62–118 days) from date of IORT. Although the intent of the study was to administer IORT as a boost to EBRT, four patients did not receive EBRT; one patient received brachytherapy, two patients declined EBRT, and one patient died of unrelated causes before receiving EBRT. The patient who received brachytherapy in addition to IORT had an unusual dumbbell-shaped tumor and his treatment differed somewhat from the standard approach at our institution. The superficial portion of the tumor was completely resected and received IORT; the deep portion of the tumor proved unresectable at the time of surgery and was therefore treated with brachytherapy.

Patient follow-up and clinical outcomes are summarized in Table 2. Patients 1–8 experienced disease recurrence. Patient 1 failed within the IORT field, but died of metastatic disease 3 months after surgery and IORT. Patient 2 failed adjacent to the IORT field and is currently alive with disease (AWD). Patients 3–8 failed at distant sites, with five patients developing lung metastases and one patient having retroperitoneal lymph nodes involvement. None of the 6 patients with positive surgical margins had a local recurrence. Three patients presented with metastatic disease at the time of diagnosis (patients 4, 7, and 8). Patient 4 is AWD 17 months after diagnosis, and patients 7 and 8 died of metastatic disease, 5 and 41 months after diagnosis, respectively, both with control of local disease sites.

**Figure 1:**
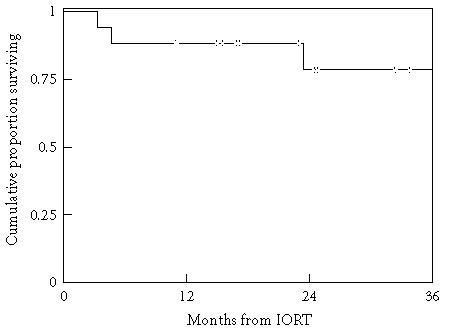
Overall survival. Kaplan-Meier probability estimates. Four of the 17 patients died.

**Figure 2:**
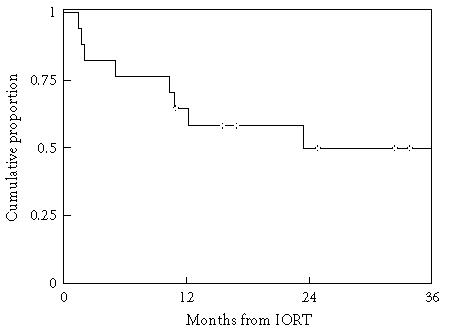
Disease-free survival. Kaplan-Meier probability estimates. Two patients failed locoregionally, 6 patients failed distantly, and 1 patient died without recurrence.

**Figure 3:**
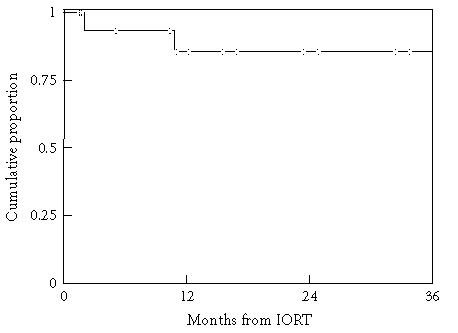
Locoregional control. Two of the 17 patients failed locoregionally.

Median follow-up for all patients was 23 months (range 3–53 months), with median follow-up for surviving patients of 24 months (range 11–53 months). Median OS was 41 months but only 1 patient has been followed beyond the median time indicating that this estimate may reflect censoring (Figure 1). Eight patients are alive with no evidence of disease (NED). One patient died of unrelated causes prior to initiation of EBRT, while still NED. Median DFS was 23.4 months, resulting from 2 locoregional failures, 6 distant failures, and 1 death without recurrence (Figure 2). Two of 17 patients failed locoregionally at 2 and 11 months after IORT (Figure 3). The median has not been reached for locoregional control, but locoregional control remains at 86% as of 11 months after IORT with the longest failure-free observation being 44 months. Thirty-six month estimates for locoregional control, DFS, and OS, were 86%, 50%, and 78%, respectively.

Of the 17 patients, five were 18 years of age or younger at diagnosis. Three of the five pediatric patients had positive surgical margins following GTRs. None of the pediatric patients recurred locally. Patient 5 developed retroperitoneal lymph node metastases and is currently AWD. Of patients 3, 7, and 8 who developed lung metastases, two have died of disease and one is AWD. Patient 9, who presented with localized synovial sarcoma of the forearm, is alive, without evidence of disease, 39 months after resection with IORT.

IORT was extremely well tolerated in our cohort. Although follow-up was not long enough to assess late toxicities associated with IORT, there were no acute toxicities noted. Of the 13 patients who received subsequent EBRT, six experienced mild to moderate erythema, one experienced pain, one blistering, and three patients experienced desquamation.

## DISCUSSION

STSs are characterized by high local recurrence rates following surgical excision alone. While the addition of radiation therapy has successfully improved local control, multimodality therapy for extremity sarcomas has sustained local control rates while limiting the need for amputations and morbid surgical procedures. The benefits of radiation therapy for STSs have been documented in retrospective as well as prospective, randomized trials [13, 24]. However, delivering adequate doses of EBRT to extremity lesions can present particular challenges.

Complications including wound breakdown, skin graft failure, and flap necrosis occur in approximately 17% of patients receiving postoperative EBRT and 35% of patients receiving preoperative EBRT [24–26]. In addition, extremities are particularly susceptible to long-term sequelae of fibrosis and edema resulting from EBRT administered in the preoperative or postoperative setting [24]. We therefore sought to enhance the benefits of EBRT, while reducing associated toxicities by utilizing an IORT boost for patients with extremity STSs.

In the treatment of sarcomas, IORT has generally been used for tumors arising in the abdomen and pelvis. In a prospective trial of retroperitoneal sarcomas, 35 patients were randomized between 50–55 Gy of EBRT alone and 20 Gy IORT plus 35–40 Gy EBRT. Although initial analyses demonstrated less toxicity in the IORT arm without improvement in DFS or OS [27], an updated analysis revealed a significant reduction in locoregional recurrences in the group receiving IORT [28]. This group also had a lower incidence of disabling enteritis, although peripheral neuropathy was more common in the IORT group than among controls receiving high-dose EBRT without IORT. A more recent phase I trial of patients with localized retroperitoneal sarcomas demonstrated that IORT was feasible and was successfully administered [22].

Few studies address local control, tissue tolerance, and effectiveness of IORT in the treatment of extremity STSs. Azinovic et al reported a study of 45 patients with extremity STSs treated with IORT [29]. They reported 5-year local control rates of 88% in patients with negative surgical margins and 57% in patients with positive surgical margins. Five patients developed neuropathies following treatment. Dubois et al published a series of 31 patients with STSs, of which 18 patients had extremity STSs treated with IORT [30]. They reported 4 local failures among the 31 patients but no local failures in patients with extremity or trunk STSs.

Van Kampen et al treated 68 extremity STS patients with IORT and reported a 5-year OS of 70% and local control rate of 88% [31]. Analyzing a subset of 58 patients for late sequelae using the LENT-SOMA scoring system for soft-tissue fibrosis, they noted that 4 patients had Grade 1-, 2 patients had Grade 2-, 5 patients had Grade 3-, and 1 patient had Grade 4-fibrosis. Lehnert et al reported a series of 251 patients with soft-tissue sarcomas; 131 patients had extremity STSs, of which 55 received IORT [32]. Five-year OS and local control rates were 78% and 83%, respectively, with a surgical complication rate of 25%. Although treatment assignment to IORT was nonrandom, it is interesting to note that among patients who did not receive IORT, the 5-year OS rate was 57% and the 5-year local control rate was 68%, reflecting a trend toward decreased morbidity and mortality with the use of IORT.

Given these preliminary, yet promising experiences of IORT in the treatment of STSs, we sought to explore our institutional experience using IORT as a component of multimodality therapy for extremity STSs. Of these patients, 17 had extremity STSs that were treated with IORT and the locoregional control was 86%, a figure comparable to prior published results. There were no locoregional recurrences in the 6 patients with positive surgical margins. Previous studies have shown a lower local regional control rate in patients with positive surgical margins [1, 29], but our results suggest that IORT as a boost could represent an improvement in the management of these patients. In addition, EBRT administered to the extremities poses particular challenges with significant risks of fibrosis, edema, and compromised function. IORT was well tolerated in our cohort with no acute toxicities, but follow-up was not long enough to comment on late toxicities such as neuropathy or fibrosis. Thirteen patients experienced various degrees of dermal toxicities following EBRT, none of unexpected severity.

Although we present encouraging results, the report is limited by the retrospective nature of the study. It includes a small number of patients, diverse histologies, various stages of presentation, and multiple treatment techniques. However, given these hopeful results, we anticipate a future prospective study that will address these limitations. In such a future study, we hope to demonstrate the utility of IORT as a boost to EBRT and a means of providing excellent local control with limited acute toxicity. It is our institution's policy to use IORT as a boost to EBRT for all patients with soft-tissue sarcomas of the extremity in whom proximity to critical structures such as neurovascular bundles precludes complete resection with adequate, negative margins.

## Figures and Tables

**Table 1:** Patient characteristics and treatment details.

Variables	Number

Number of patients	17
Median age (range), years	37 (8–86)
Female : male	8 : 9

Primary tumor location	

Upper distal extremity	4
Upper proximal extremity	1
Lower distal extremity	3
Lower proximal extremity	9

Histology	

Synovial sarcoma	6
Malignant fibrous histiocytoma	4
Alveolar rhabdomyosarcoma	2
Ewing sarcoma	2
Angiomyxoma	1
Angiosarcoma	1
Leiomyosarcoma	1

Stage (AJCC)	

I	1
II	6
III	7
IV	3
EBRT, yes : no	13 : 4
Chemotherapy, yes : no	10 : 7
Surgical margins,* positive : negative	6 : 11

Disease status at time of IORT	

Primary	16
Recurrent	1

*Abbreviations*: AJCC = American Joint Committee on Cancer. IORT = intraoperative radiation therapy. EBRT = external beam radiation therapy. *A positive surgical margin was defined as a margin less than 1 mm.

**Table 2:** Patient outcome and follow-up.

Pt	Age at Dx	Histology of primary	Location of primary	Presenting stage	Failure in IORT field	IORT dose (Gy)	Site of EBRT	Surgical margins	Site of first progression after IORT	Status at last FU	FU (months)

1	86	Malignant fibrous histiocytoma	Buttock	III	Yes	12.5	Buttock	−	Buttock	Dead	3
2	68	Malignant fibrous histiocytoma	Thigh	III	No	12	Thigh	−	Acetabulum	AWD	23

3	18	Synovial sarcoma	Knee	II	No	15	Knee	−	Lungs	AWD	15
4	39	Synovial sarcoma, recurrent	Thigh	IV	No	15	Thigh	−	Lungs	AWD	17
5	9	Alveolar rhabdomyosarcoma	Calf	III	No	12	None	−	RP LN	AWD	53
6	54	Angiosarcoma	Thigh	II	No	15	Thigh	+	Lungs	AWD	24
7	9	Ewing sarcoma	Prox add	IV	No	12.5	Prox add	+	Lungs	Dead	5
8	18	Ewing sarcoma	Buttock	IV	No	12.5	Buttock	+	Lungs	Dead	41

9	8	Monomorphic synovial sarcoma	Forearm	II	No	12.5	Forearm	+	None	NED	39
10	76	Malignant fibrous histiocytoma	Groin	III	No	15	Groin/add	−	None	NED	15
11	59	Malignant fibrous histiocytoma	Forearm	III	No	12.5	Forearm	+	None	NED	32
12	31	Angiomyxoma	Thigh	I	No	15	None	−	None	NED	37
13	20	Alveolar rhabdomyosarcoma	Forearm	III	No	12.5	Forearm	−	None	NED	17
14	21	Synovial sarcoma	Shoulder	II	No	12.5	None	−	None	NED	11
15	46	Synovial sarcoma	Hand	III	No	12.5	Hand	+	None	NED	34
16	37	Synovial sarcoma	Popl fossa	II	No	12.5	Popl fossa	−	None	NED	25
17	38	Leiomyosarcoma	Groin	II	No	12.5	None	−	None	Dead	23

*Abbreviations*: Pt = patient number; Dx = diagnosis; IORT = intraoperative radiation therapy; EBRT = external beam radiation therapy; FU = follow-up; prox = proximal; add = adductor; popl = popliteal; RP = retroperitoneal; LN = lymph node; AWD = alive with disease; NED = no evidence of disease.

*Note*: Patients 1–2 failed locoregionally and patients 3–8 failed distantly.
